# Phytotherapy for the Treatment of Glioblastoma: A Review

**DOI:** 10.3389/fsurg.2022.844993

**Published:** 2022-03-25

**Authors:** Shan Yasin Mian, Abhishek Nambiar, Chandrasekaran Kaliaperumal

**Affiliations:** ^1^Department of Surgery and Cancer, Imperial College London, London, United Kingdom; ^2^College of Medicine and Veterinary Medicine, University of Edinburgh, Edinburgh, United Kingdom; ^3^Department of Clinical Neurosciences, Royal Infirmary of Edinburgh, Edinburgh, United Kingdom

**Keywords:** phytotherapeutic agent, glioblastoma multiforme, glioblastoma, alternative, extract

## Abstract

**Background:**

Phytotherapy; the study of extracts of natural origin in the treatment of disease, has scarcely been applied in the management of GBM. A body of literature exists studying *in-vitro*, the use of natural extracts against GBM cells. Given persisting poor prognoses, we evaluated, through systematic literature-review the therapeutic potential of naturally sourced extracts *in-vivo*.

**Methods:**

Using OVID, MEDLINE and EMBASE databases were searched with compound search term. Abstracts and full-texts were double-screened by independent reviewers.

**Results:**

Nine hundred and eighty-seven articles, excluding duplicated were screened, leading to the inclusion of 14. Amongst murine studies, Ashwagandha, Coptis Chinensis and Fructus Ligustri Lucidi in unprocessed forms, produced significant reductions in tumour volume. Amongst human studies, Perrilyl alcohol, derived from Lavender, reduced angiogenic cytokines in 31% of subjects, halted 6 month disease progression in 48.2% of subjects, and improved mean survival by 4.9 months in separate studies, respectively.

**Conclusion:**

Although cursory, current trends in literature demonstrate the value of inhaled Lavender extract in the treatment of GBM, offering tangible clinical benefit to patients receiving conventional treatments. Furthermore, the administration of 8, discrete extracts in mice to produce significant responses in survival and tumour volume, suggest there is further scope for study. Although additional safety tests are required, currently, phytotherapeutics are the crossover to clinical translation, and additional trials are warranted to expound upon thus far promising results.

## Introduction

In the domain of intra-cranial malignancies, Glioma, in particular the WHO Grade IV Glioblastoma, persists as having one of the worst prognoses in modern medicine. Despite developments with the Stupp protocol leading to the convention of surgery with chemo-radiotherapy as the mainstay of treatment, mean survival rarely exceeds 18 months (1). Since the Stupp protocol was first devised, treatment for Glioblastoma has remained relatively static globally, and outcomes improved little over the years (2, 3).

As with other phytotherapeutic success stories, such as digitalis to digoxin (4) or the nightshade plant to atropine (5), there is a body of literature documenting the search for a compound of botanical origin with treatment potential. Several reviews in recent years demonstrate the identification of compounds including terpenes, flavonoids and cannabinoids, originating from a variety of plant species (6–8). Evidence demonstrates a significant response of these compounds against Glioblastoma in both *in-vitro* and *in-vivo* models. As with non-synthetic compounds, the study of these extracts and possible translation to clinical practice may take many years; and many may not have any discernible impact upon clinical outcomes despite *in-vitro* success. In this mini-review, we instead seek to summarise the evidence concerning the therapeutic potential of non-invasively administered compounds of natural origin for Glioblastoma *in-vivo* that have not been processed, using a cursory literature search to compile the most prominent active compounds with the potential to be used, and that are currently undergoing research.

Historically, the benefits of non-invasively administered phytotherapeutics have been evident insofar that they have formed the majority of medical practice in the pre-modern age. Ayurvedic, traditional Chinese medicines and other complimentary therapies often use exclusively herbal remedies to target disease and remain prevalent in many societies today (9). The relative affordability and ease of obtaining non-synthetic remedies, as well as the generally muted side effect profile associated with their administration, mean that the overall risks associated with the use of these medications are relatively low (10). If an evidence base can establish that such treatments are significantly therapeutic, it would undoubtedly merit their more frequent use and exploration. Efficacious natural compounds have the potential to make a tangible difference to the disease course and symptomatic control of countless victims of Glioblastoma across a broad variety of health setting, in particular those that are resource poor. Where possible, we aimed to thus summarise the key agents and enable healthcare practitioners and surgeons to access the evidence surrounding them, so that this can be shared with patients who may ultimately elect to use them to supplement their conventional therapies.

In service of these aims, we devised the following key objectives:

- Determine whether there is current evidence available to support the use of a phytotherapeutic extract in the treatment course of Glioblastoma.
- Evaluate and compile the current body of literature on the *in-vivo* use of phytotherapeutics extracts, specifically for Glioblastoma.
- Postulate extracts of particular note which may merit further research or are likely to undergo clinical translation in the near future.

## Methodology

Our literature search was kept in adherence to the Prisma P protocol for the reporting of systematic literature reviews section on searching (11). The search itself was conducted using the OVID platform, searching the Medline (commonly known as Pubmed) and Embase databases. We constructed a broad search term, individualized to each database with liberal use of MeSH exploded terms, to include as wide a variety of articles as possible, with the proviso that it only considered those with significant findings. It was devised after meticulous evaluation of seminal articles on phytotherapy and Glioblastoma, as well as exploratory searches to ascertain the most common key terms that recur throughout the literature, to ensure that only the most seminal and pertinent articles would be compiled.

The individualized search terms were as follows:

Database 1: Ovid MEDLINE(R) ALL <1946 to January 08, 2021>

Search Strategy:

1. glioblastoma^*^.mp. (44741)
2. glioma/ or glioblastoma/ (63644)
3. 1 or 2 (76953)
4. phytotherapy.mp. or Phytotherapy/ (39711)
5. Plant Oils/ or essential oil^*^.mp. or Plants, Medicinal/ or Plant Extracts/ (191856)
6. terpenes/ or cannabinoids/ (24162)
7. Flavonoids/ (40888)
8. Phenols/ or Phenol/ (54271)
9. 4 or 5 or 6 or 7 or 8 (298646)
10. 3 and 9 (594)

Database 2: Embase Classic+Embase <1947 to 2021 January 11>

Search Strategy:

1. glioblastoma^*^.mp. (83492)
2. glioma/ (62351)
3. 1 or 2 (129831)
4. phytotherapy/ (18049)
5. medicinal plant/ (87613)
6. terpene derivative/ or terpene/ or terpene.mp. (20786)
7. cannabinoid/ (12539)
8. flavonoid/ (59961)
9. phenol/ (31627)
10. 4 or 5 or 6 or 7 or 8 or 9 (210573)
11. 3 and 10 (480)

Following the initial search, automated and manual deduplication was actioned and two independent reviewers conducted an abstract screening with the following inclusion criteria:

- All articles must consist of original evidence, no review or meta-analysis articles were considered
- *In-vivo* evidence only was considered, exclusively *in vitro* studies were excluded. Both human and non-human experimental models were considered.
- Interventions must have consisted of non-synthetic isolates or fractions, from naturally sourced botanicals. Excessive processing or chemical reconfiguration of extracted compounds was not permitted and these articles were excluded.
- All interventions must have been administered non-invasively.
- A control arm was not necessary.
- Any outcome relating to the disease course, and therapeutic success of and against GBM was considered.

Where disagreements arose, they would be settled through consensus with a third reviewer. The reference lists were then extracted from the included full text. Articles that cited them were compiled through the SCOPUS citation search function. These underwent the same abstract screening as above to yield the final list of full-text articles to be screened.

These full-text articles would be screened with the above inclusion criteria in mind, and those excluded done so with agreed reasons. Disputes would be settled through our third reviewer in consensus. After the final screening, data was extracted and tabulated through Excel. Although sufficient data to warrant statistical meta-analysis was not anticipated, data categories that would be conducive to comparison were extracted. In addition to subject demographics, the specific population and intervention types, as well as key data surrounding the type of outcome was sought. Where data was missing, authors were contacted to request full release.

A key “headline” conclusion from the data of each article was extracted in service of our goal of compiling a variety of interventions that target the different symptomatic faculties of GBM. Finally, where murine or other large mammal studies were considered, any articles that did not comply with local ethical approval and animal management guidelines were not included.

## Results

Our search, after automated and manual deduplication, yielded 987 articles that underwent abstract screening. This included all manual additions from reference lists and citation search through SCOPUS of included abstracts. Twenty Articles had their full texts read, and of those 14 were selected for inclusion, with nine studies using murine models and five using human subjects. No discrepancies were noted between reviewers and, where possible, full datasets and English translations of original works were sought before inclusion in the review.

A variety of outcomes were reported; in most articles, survival and a measure of tumour size (e.g., post-mortem volume, size on imaging, or fluorescent signal) were the metrics of choice by which to judge intervention effect. Several murine studies also reported cell proliferation and differentiation, as judged on tumour pathology. Concerning study demographics, reporting was significantly lacking, with numerous trials failing to report the number of subjects, period of study, or per subject data points. A number of these trials were reported as abstracts only and were not published thereafter; attempts at obtaining further data was unsuccessful.

The human-based trials most commonly tested three different agents. Intranasal Perrilyl Alcohol, obtainable from a variety of sources including lavender, sage, and lemongrass, was used in three trials with successful effect. In the first 6 months after diagnosis, administration was noted to significantly increase survival across all subjects (numbering 142 across the two trials that reported this outcome), with no disease progression in nearly half of participants in Fonseca et al's. 2009 study. The reduction in inflammatory cytokine circulation was not accompanied by an outcome reporting survival in the same subjects, and this paper stands alone in reporting this outcome.

Other studies using human subjects, included the use of oral Nairingin sourced from citrus extract to alleviate post-craniotomy pyrexia and associated inflammation in GBM patients, with a significant reduction noted in all patients trialed.

The final human trial was a retrospective large cohort study that evaluated whether caffeine (as taken in coffee) had any effect on the risk of GBM. This study of over half a million participants noted a significant, dose-dependent reduction in risk in those who consume over 100 ml of coffee per day, accounting statistically for all other variables. Given the near absolute heterogeneity of outcomes, with all studies reporting different measures, even for variables like survival, further statistical analysis was not feasible.

A similar array of outcomes was reported for the murine studies. Amongst the 9 included, mean survival was the most reported outcome; however, a significant number of studies reported tumour volume, as well as level of cell proliferation and differentiation, given the added benefit of widely available pathology specimens in these trials.

All mice were given the investigated phytotherapeutics through oral gavage using a variety of extracts. Notably, several trials such as Li 2017, Jeong 2011, and Bentrad 2015 utilized unprocessed, raw herbs as part of their treatment protocol. All reported significant improvement in survival and reduction in tumour volume, often on a dose-dependent basis.

Of the included murine articles, all reported significant improvement in survival or tumour volume/differentiation except for one article. Ham 2019, employing Ginseng extract demonstrated no significant improvement, with only modest effects on tumour differentiation noted. No two studies utilized the same intervention extract. In general, studies did not expound on the details of control arms or subject demographics. Consequently, no further statistical analysis was considered. Tables 1, 2 summarizes the study characteristics of literature that included human and murine models respectively.

**Table 1:** Summary of study characteristics of included articles examining human models.

**Study**	**Intervention**	**Mode of administration**	**Outcome**	**Presence of control**	**Intervention sample size**	**Result**
Fonseca et al. (2011)	Perrilyl Alcohol	Intranasal	Mean survival	52 untreated subjects	89	7.2 mean survival in treated group, as opposed to 2.3 in control.
Fonseca et al. (2009)	Perrilyl Alcohol	Intranasal	Halt of Progression after 6 months	No controls	52	48.2% of intervention group experienced no disease progression at 6 months
Gomes et al. (2011)	Perrilyl Alcohol	Intranasal	Compound of significant reduction in circulating angiogenic cytokines	Compared to historical literature controls	83	31% of subjects displayed a significant reduction in angiogenic cytokine levels
Fan et al. (2001)	Nairingin (Citrus Extract)	Oral	Mean number of febrile episodes post-craniotomy after 7 days of treatment	Compared to historical literature controls	not specified	Subjects treated with Nairinging demonstrated significant reduction in number of pyrexial episodes
Michaud et al. (2010)	Coffee	Oral	Risk of GBM development in retrospective cohort study	Compared to baseline population risk	5,21,448 (Total participants in cohort)	Significant correlation between increased coffee consumption (100 ml daily) and a reduction in risk of developing GBM

**Table 2:** Summary of study characteristics of included articles examining murine models.

**Study**	**Intervention**	**Mode of administration**	**Outcome**	**Result**
Bentrad (2015)	Trigonella foenum graecum (unprocessed abstract)	Oral	Survival, growth of tumour, volume of metastases	Mean lifetime prolonged by 15-50%, with growth of index tumour reduced by 25-48% and mean volume of metastases reduced by 18-86% in treated group
Cao et al. (2016)	Toosendanin	Oral	Mean tumour size	Significant decrease in tumour size and weight in all treated group mice
Chang et al. (2016)	Ashwagandha (cherry tree extract)	Oral	Intensity of biofluorescence of tumour tissue	Significant reduction in biofluorescent signal in all treated mice
Ferreira et al. (2016)	Casearin X (Casearia sylvestris Swartz)	Oral	Growth rate of GBM across remaining murine lifespan	Dose dependent inhibition of growth rate up to 67.4% compared to non-treated control
Ham et al. (2019)	Ginsenoside (Panax ginseng)	Oral	Survival, and mean level of cellular proliferation seen in pathology	No significant change to survival, some reduction in cellular proliferation seen but not statistically significant
Hilliard et al. (2017)	15alpha-methoxypuupehenol (Hyrtios)	Oral	Tumour volume and TUNEL assay	Significant reduction in tumour volume in all treated mice, and a significant reduction in DNA fragmentation TUNEL scores in all treated mice
Jeong et al. (2011)	Fructus Ligustri Lucidi (unprocessed extract)	Oral	Tumour volume	Significantly reduced tumour volume in intervention group
Jeong et al. (201)2	Apigenin(Lycii cortex radicis)	Oral	Proliferation rate (pathology) and Tumour volume	Dose dependent reduction in tumour volume and cell proliferation rate and tumour volume in intervention group
Li et al. (2017)	Coptis Chinensis (unprocessed extract)	Oral	Survival and mean tumour volume	Low doses achieved significant improvement in survival, and decrease in tumour volume

## Discussion

Our review of the literature aimed to evaluate the growing trends in phytotherapy for the treatment of GBM. To that effect, murine and human studies have given us some insight. Firstly, concerning the human studies, Perrilyl Alcohol achieved some success in recent human trials, with significant survival benefit. Found in lavender and certain citrus fruits, its extracted use has been researched previously for other malignancies (12). The evidence base for its use in GBM is growing; recent registrations of trials aim to expand the evidence for a variety of GBM mutations. Some centers have already begun preliminary use for alleviation of symptoms from mass tumors in Glioblastoma patients.

Belonging to the Monoterpene class of compounds, Perrilyl Alcohol has previously been the subject of *in-vitro* trials (7). Later studies noted nauseating effects when administered orally. Thus, intranasal administration could represent an exciting new avenue for adjuvant treatment, as indicated by preliminary indications of safety and efficacy.

Other human trials offered less directly therapeutic, but equally interesting results. The use of Naringin, often sourced from grapefruit, demonstrated a significant reduction in postoperative pyrexia after surgery for GBM. Pyrexia is correlated to a worse prognosis post-operatively, with greater susceptibility to infection and overwhelming inflammation (13). Besides, it is profoundly uncomfortable for patients; thus managing pyrexia comprises a fundamental part of symptomatic relief.

Furthermore, although it falls outside the scope of our study, some excluded articles mentioned the value of sulforophane (14, 15), commonly found in cruciferous vegetables in reversing the resistance of GBM cells against temozolomide. Although this again does require combination with conventional therapies and is not evidenced as a standalone extract, as an avenue of research for future studies it provides a possible domain of alternative and combined exploration.

The retrospective cohort study concerning coffee was the largest included in our review. The evidence generated concluded that over 100 ml of standard coffee consumption every day reduced, on a dose-dependent basis the risk of GBM and other Gliomas was very promising.

Despite these promising results, translation into clinical benefit remains limited by some significant confounding variables. All interventions were deployed in patients treated by conventional means. Consequently, conventional treatment was a major confounding factor when assessing phytotherapeutic efficacy. Given the severity of Glioblastoma, ethical justification for superiority studies would be tenuous. Furthermore, the adjuvant nature of introduced agents means there was little attention paid to control groups or confounding variable control.

In the murine trials, some of these limitations were not applicable. Across the board, no negative results were found in the included articles; all bar one reporting significant benefit of the trialed phytotherapeutic. A wider variety of agents was also used, with extracts such as Ashwagandha (cherry tree root) and Coptis Chinensis, used, respectively in traditional ayurvedic and Chinese herbal medicines. More significantly, a number of these extracts were administered without any processed extraction. Giving these herbal remedies in their raw form still produced significant results and favors similar administration in humans. Efficacy in raw form further bolsters ease of access and affordability for patients due to the elimination of unnecessary processing and refinement.

Murine studies also considered the effect of tumour volume, level of cell differentiation, and gross number of metastases following phytotherapeutic treatment. Although these outcomes are not definitive markers of survival, they may act as a surrogate for patient comfort. Limiting tumour size and metastatic/differential potential can significantly impact the symptomatic burden of Glioblastoma. Larger tumors often incur greater mass effect and present with increased pain and a lowering of seizure threshold (16). Therefore, even if life expectancy remains unaffected, the symptomatic benefit of phytotherapeutics has the potential to be significant.

The major question this evidence raises is whether adjuvant phytotherapy can be implemented into clinical practice. Although our evidence would suggest this is safe to do, there are some limitations. As broad search terms were purposefully employed and given the prevalent use of phytotherapeutics and traditional therapies in Asian countries, an inestimable number of articles will not be indexed in English language databases.

Conclusions on the use of combinations of extracts and efficacy are also difficult to draw from our review which concentrates largely on sole extracts. All included articles trialed single extract therapies only. Although a variety of phytotherapeutic agents were associated with anti-GBM effects with relative safety, suggesting that combinations may have the potential to be effective.

Another interesting trend seen is that most consequential phytotherapeutic literature on GBM has only been reported over the last decade. Given that herbal extracts have been in use for millennia, it is interesting that their use has not been explored priory, as digitalis had been in cardiology for example. This may be attributed to increased diagnostic rates and prevalence of Glioblastoma recently or an increased awareness of herbal medicine. Alternately, it may be a consequence of reluctance to trial herbal extracts and traditional therapies in the modern era. Often, they are dismissed as pseudo-science or considered to detract from conventional therapies. Indeed, if the evidence demonstrates the efficacy of a non-synthetic extract in providing partial therapeutic value in GBM, it may dissuade patients from adherence to conventional chemo-radiation and surgical treatments. However, the multifactorial anthropological factors influencing this behavior are beyond the scope of this article.

Despite the above reservations, intranasal Perrillyl Alcohol appears to be the first phytotherapeutic with a developing body of evidence demonstrating efficacy and safety in human trials. It is safe to conclude that it may start to be clinically translated soon, with implementation into the management of GBM worldwide. Furthermore, a host of promising extracts from the Terpene and Flavonoid groups, as well as unprocessed herbs such as Ashwagandha are demonstrating good effect against tumour sequelae in murine models. Other compound groups such as cannabinoids which have shown effectiveness *in-vitro* yet remain to be applied *in-vivo* and offer avenues for future study. No safety concerns or side-effects have thus far been raised in the literature, corroborating pre-existing safety profile claims.

The phytotherapeutics evidence base is beginning to encroach upon clinical practice. Pre-established stigma poses a major barrier to further research. Our mini-review demonstrates that the emphasis on these alternative phytotherapeutics, should be that they may be a helpful way to alleviate symptoms associated with GBM, and can be used in tandem with conventional therapies rather than as a replacement to them. While herbal remedies are not frankly curative, their expanded use may offer novel, cheap and accessible avenues in the continued struggle against this difficult disease.

## Author Contributions

SM conducted the review, with AN acting as second reviewer. SM, AN, and CK wrote the manuscript, with CK supervising and mediating the review. All authors contributed to the article and approved the submitted version.

## Conflict of Interest

The authors declare that the research was conducted in the absence of any commercial or financial relationships that could be construed as a potential conflict of interest.

## Publisher's Note

All claims expressed in this article are solely those of the authors and do not necessarily represent those of their affiliated organizations, or those of the publisher, the editors and the reviewers. Any product that may be evaluated in this article, or claim that may be made by its manufacturer, is not guaranteed or endorsed by the publisher.
